# Vestibular-Somatosensory Interactions: Effects of Passive Whole-Body Rotation on Somatosensory Detection

**DOI:** 10.1371/journal.pone.0086379

**Published:** 2014-01-21

**Authors:** Elisa Raffaella Ferrè, Mariia Kaliuzhna, Bruno Herbelin, Patrick Haggard, Olaf Blanke

**Affiliations:** 1 Institute of Cognitive Neuroscience, University College London, London, United Kingdom; 2 Laboratory of Cognitive Neuroscience, Brain Mind Institute, École Polytechnique Fédérale de Lausanne, Lausanne, Switzerland; University of Cardiff, United Kingdom

## Abstract

Vestibular signals are strongly integrated with information from several other sensory modalities. For example, vestibular stimulation was reported to improve tactile detection. However, this improvement could reflect either a multimodal interaction or an *indirect* interaction driven by vestibular effects on spatial attention and orienting. Here we investigate whether natural vestibular activation induced by passive whole-body rotation influences tactile detection. In particular, we assessed the ability to detect faint tactile stimuli to the fingertips of the left and right hand during spatially congruent or incongruent rotations. We found that passive whole-body rotations significantly enhanced sensitivity to faint shocks, without affecting response bias. Critically, this enhancement of somatosensory sensitivity did not depend on the spatial congruency between the direction of rotation and the hand stimulated. Thus, our results support a multimodal interaction, likely in brain areas receiving both vestibular and somatosensory signals.

## Introduction

Vestibular signals contribute to several complex behaviours and cognitive functions and are integrated with inputs from other sensory modalities. For example, the vestibular system plays a key role in spatial orientation and self-motion detection. Consistent with this view, functional neuroimaging studies in humans revealed that vestibular inputs project to a network of subcortical and cortical multimodal areas, particularly to the posterior insula and adjacent operculum [1], [2], [3], [4], [5].

Critically, the vestibular cortical projections strongly overlap with the somatosensory cortical projections [4], [5], [6], [7]. There is growing evidence for multisensory perceptual interactions between vestibular and somatosensory signals. Both caloric and galvanic vestibular stimulation (CVS, GVS respectively) were shown to modulate tactile perceptual thresholds [8], [9], and somatosensory-evoked potentials (SEPs) [10]. In particular, CVS selectively enhanced the N80 SEPs wave [10], whose source has been localised in the parietal operculum [11], [12]. Clinical studies showed that CVS and GVS produce transient remission of hemianaesthesia in brain-damaged patients [13], [14], [15], [16].

**Figure 1:**
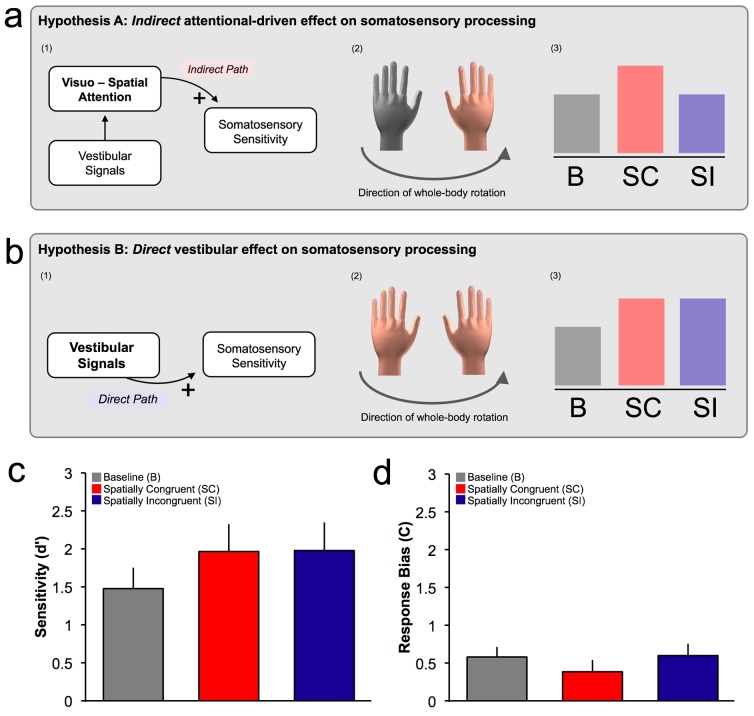
Experimental hypotheses and results. Experimental hypotheses are illustrated in panel (a) and (b). (a1) Somatosensory processing might be modulated by an *indirect* attentionally-mediated (spatially-selective) effect of vestibular stimulation. An indirect effect on somatosensory processing predicts an improved tactile sensitivity only when touch and rotation are spatially congruent, e.g., touch on the right hand and rotation toward the right (a2). In particular, the indirect effect (a3) would induce no improvement in tactile sensitivity between a no rotation Baseline condition (B) and Spatially incongruent condition (SI), but a selective enhancement of sensitivity in the Spatially congruent condition (SC). Alternatively, (b1) somatosensory processing might be directly (non spatially-selective) influenced by vestibular signals. This predicts an enhancement of tactile sensitivity independent of the spatial relation between location of touch and direction of rotation (b2). In particular, this effect (b3) would not predict differences in sensitivity between Spatially congruent condition (SC) and Spatially incongruent condition (SI), critically it predicts that both conditions (SC and SI) would be different compared to Baseline condition (B). (c) Sensitivity (d’) data as a function of experimental conditions. d’ estimates support the hypothesis of a direct vestibular induced modulation. (d) Response bias (C) data as a function of experimental conditions.

However, both CVS and GVS involve unnatural peripheral stimulation. They activate not only classically ‘*vestibular*’ and multisensory areas, but also attentional and visuo-spatial processing regions [5], [17]. Thus, at least two possible mechanisms could underlie vestibular-somatosensory interactions observed with CVS and GVS. First, vestibular stimulation might modulate somatosensory processing, for instance via neurons receiving both vestibular and somatosensory signals (Figure 1b) [10]. Alternatively, vestibular stimulation might influence somatosensory perception *indirectly*, via a supramodal spatial attentional mechanism [13], [14] (Figure 1a). This alternative indirect hypothesis is plausible given the strong effects of vestibular inputs in orienting of spatial attention [18] and in orienting behaviours generally [19]. Thus, it is important to identify whether vestibular effects on the somatosensory system are spatially-selective or not.

Studies describing the vestibular induced modulation of tactile processing suggested a direct vestibular interaction with somatosensory circuits [8], [9], [10]. Interestingly, the changes in the somatosensory thresholds and sensitivity were found on both left and right hand following unilateral vestibular activation. However, previous experiments cannot rule out additional non-specific effect of vestibular stimulation on somatosensory processing, because of the strong effects of artificial vestibular stimulation. For example, vestibular stimulation could influence performance because of general arousing effects, or by shifts of spatial attention. The former hypothesis has been ruled out by a number of studies using artificial vestibular stimulation to compare generic effects, resulting from stimulation of either hemisphere, to hemisphere-specific effects obtained with particular lateralisation of stimulation [15], [16]. For example, it has been recently reported that hemisphere-selective left anodal and right cathodal polarity of GVS significantly enhanced sensitivity to mild shocks on either hand, while no such effect was found with either right anodal and left cathodal GVS or sham stimulation [9]. These hemisphere-specific effects cannot readily be explained by general arousal, since the peripheral vestibular organs receive comparable stimulation in both cases. In contrast, artificial vestibular stimulation studies are less able to rule out accounts based on spatial attention, because they cannot precisely control the spatial aspects of the stimulation.

Here we investigated the vestibular enhancement of tactile processing using a *natural* activation of vestibular cortical projections, through passive whole-body yaw rotation. This method of stimulation permits precise control over the spatial signals generated in the peripheral vestibular system, and therefore over spatial congruency/incongruency. It allowed us to test whether vestibular effects on tactile perception were present also with this more natural stimulation, and whether they were spatially-selective or not.

Spatially-selective perceptual mechanisms have been reported in neurons responding to visuo-auditory [20], visuo-tactile [21], and visuo-vestibular stimuli [22], [23]. For instance, stimuli signalling motion in complementary directions (e.g. vestibular rotation to the right and optic flow to the left) are preferentially integrated. Multisensory neurons coding for visual, vestibular and somatosensory stimuli were found in the macaque ventral intraparietal area (VIP) [24], homologous to human vestibular areas in the posterior parietal cortex [17]. Importantly, the firing patterns of the majority of VIP neurons showed a preference for ipsiversive stimuli: for instance, a bimodal neuron receiving both tactile and vestibular signals would preferentially responds to rightward rotation and to touch on the right cheek. One could thus imagine that the processing of tactile stimuli will be facilitated when coupled with vestibular rotation in the same direction.

A somatosensory detection task was administered during passive whole-body rotations. Participants sat on a rotating chair and were instructed to detect faint tactile shocks delivered to the left or to the right hand. Direction of rotation and tactile stimulation conditions were independently randomized. This orthogonal design ensured that spatial-attentional effects of congruency/incongruency could be estimated directly, and were never predictable. Recent studies [18] revealed that passive whole-body rotatory accelerations produce spatiotopic shifts of attention in the direction of rotation (i.e, congruent or ipsiversive direction), which moreover influence tactile detection. We hypothesized that an *indirect* modulation of tactile sensitivity mediated by this shift of spatial attention would produce an improved detection for faint shocks delivered to the hand spatially congruent to the direction of rotation (Figure 1a), compared to the other hand. Alternatively, we might observe non spatially-selective effects of vestibular stimulation on somatosensory detection, independent of the spatial congruency between touch and rotation (Figure 1b).

## Methods

### 1. Ethics Statement

The experimental protocol was approved by the local ethics committee (École Polytechnique Fédérale de Lausanne) and the study was conducted in line with the Declaration of Helsinki. Participants gave written informed consent to participate in the experiment before inclusion in the experiment.

### 2. Participants

Fifteen naïve paid right-handed participants volunteered in the experiment (10 male, ages: 19–36 years, mean ± SD: 23.67±4.51 years). Handedness was assessed through informal verbal inquiry. Data from three participants was discarded due to an inability to correctly estimate reliable sensory thresholds (see below for further details).

### 3. Experimental Procedure

The experiment was conducted in complete darkness in a sound-shielded room in which a human motion platform was placed (see also [25]). Whole-body passive rotations were performed around the yaw axis by placing participants in a chair mounted on a two meters beam platform fixed on a digitally controlled electrical engine. The system (PCI-7352) operates with a precision of +/−0.1 deg/sec for a peak acceleration of 400°/s^2^ (+/−6, torque >2kNm). Participants were seated in the chair wearing seatbelts, with their head aligned to their body’s z axis and precisely located in the center of rotation. An adjustable chin-rest fixed the head position. An infrared surveillance camera monitored the subjects’ face continuously.

Participants were instructed to detect faint tactile pulses during whole-body passive rotations, and in a baseline condition involving no rotation. Since the motion platform produces slightly vibrations, the no-rotation trials were performed with motion platform powered on. This procedure insures that non specific-vestibular cues, such as vibrations of the motion platform, were equally present across experimental conditions, even if the rotator was not turning. Although the no-rotation trials controlled for platform vibrations, other bodily proprioceptive cues might be associated with whole-body rotation. Tactile electrical stimulation was delivered via a pair of ring electrodes placed over the distal phalanxes of the index fingers of both hands, with the cathode 1 cm proximal to the anode. Stimulation was delivered with a neurophysiological stimulator (Grass S48 stimulator), whose current level and pulse duration were manually controlled. To identify individual somatosensory thresholds, a staircase procedure was used to estimate the lowest shock intensity at which a tactile stimulus could be reliably detected. Independent thresholds were estimated for each participant’s left and right fingers. Pulse intensity obtained with the thresholding procedure was successively tested in a detection block and adjusted until the 40–60% of pulses were reliable detected on both fingers. This level was considered as working estimate for near threshold electrical stimulation in each participant.

Our design factorially combined passive body rotation and tactile stimulation conditions (see Table 1). Every trial involved a single rotation (if present), during which a single shock (if present) would be delivered. In particular, we were interested in three experimental conditions: (i) *Baseline condition*, in which the shock was delivered either to the left or right index finger without passive whole-body rotation; (ii) *Spatially congruent condition*, in which the shock was delivered to the hand congruent to the direction of rotation (i.e. shocks delivered to the left finger during left direction whole-body rotation and shocks delivered to the right finger during right direction whole-body rotation), (iii) *Spatially incongruent condition*, in which the shock was delivered to the hand opposite to the direction of rotation (i.e., shocks delivered to the left finger during right direction whole-body rotation or shocks delivered to the right finger during left direction whole-body rotation).

**Table 1:** Experimental conditions and stimulus design.

	Tactile stimulation	
Direction of Rotation	*Left hand*	*Right Hand*
*No rotation*	Baseline condition	Baseline condition
*Leftward rotation*	Spatially congruent condition	Spatially incongruent condition
*Rightward rotation*	Spatially incongruent condition	Spatially congruent condition

Passive body rotation and tactile stimulation conditions were factorially combined to provide independent estimates of direct vestibular modulation and indirect effects driven by factors such as attention. Every trial involved a single rotation (if present) during which a single shock (if present) would be delivered to the left or right hand.

The somatosensory detection task was designed following a signal detection approach [26]. It consisted of six tactile stimulus-present trial types: 15 trials with a shock delivered to the left hand during no rotation; 15 trials with a shock delivered to the right hand during no rotation; 15 trials with a shock delivered to the left hand during leftward spatially congruent rotation; 15 trials with a shock delivered to the right hand during rightward spatially congruent rotation; 15 trials with a shock delivered to the left hand during rightward spatially incongruent rotation and 15 trials with a shock delivered to the right hand during leftward spatially incongruent rotation. There were also six corresponding trial types in which no tactile stimulus was delivered, during the same rotation conditions. Notice that separate sets of 15 trials were used to define conditions in which no shock was delivered to the left hand and in which no shock was delivered to the right hand – this allowed separate ‘no stimulus’ trials to be used to calculate the signal detection parameters for each hand. A total of 180 trials were performed and divided in five experimental blocks. Trial order was randomized, so that participants could not predict tactile stimulus presence, hand stimulated or rotation direction. Before each experimental block a pre-test sensory detection block was administered to check the stability of the perceptual sensory threshold.

Participants were asked to fixate a white cross, centred on a 22″ computer screen mounted on the chair 40 cm in front of the eyes. The beginning of each trial was signalled by a change in the colour of the fixation cross, which became red. The rotation, if present, started after 2000 ms from the beginning of the trial. The chair’s rotation profile consisted of 1000 ms acceleration to a speed of 90°/s, followed by 1000 ms deceleration to 0°/s (raised cosine). For each block a different rotation profile was generated. The profile varied by randomized direction of rotation. The shock, if present, was delivered 2700 ms from the beginning of the trial. Thus, during the whole-body rotation trials the shock was delivered at 700 ms from the onset of acceleration, to coincide with the reported maximal firing of vestibular afferents. Participants were required to indicate whether or not they felt the shock, making un-speeded verbal responses ('yes' or 'no') during a response window of 4000 ms in which the fixation cross was green. During the experiment white noise was presented over the participants’ headphones and a black blanket covered the chair, to avoid the participant from inferring the rotation direction based on auditory or visual cues (residual light emanating from the stimulus display). Data for each trial were recorded and analysed later.

## Results

Tactile detection results were analysed using signal detection analysis [26]. The number of hits (number of tactile stimulus-present trials in which participants said ‘yes’), false alarms (number of stimulus-absent trials in which participants said ‘yes’), misses (number of stimulus-present trials in which participants said ‘no’) and correct rejections (number of stimulus-absent trials in which participants said ‘no’) was computed for each experimental condition (Baseline condition, Spatially congruent condition and Spatially incongruent condition). These values were used to obtain the perceptual sensitivity (d’) and response bias (C) estimates.

A 3x2 repeated measure ANOVA with factors of Condition (Baseline condition, Spatially congruent condition, Spatially incongruent condition) and Side of tactile stimulation (Left finger, Right finger) were performed on d’ and C estimates. Analysis of d’ values showed a just-significant effect of Condition (F(2,22) = 3.469, p = 0.049). There was no effect of Side of tactile stimulation (F(1,11) = 1.592, p = 0.233) and no interactions between factors (F(2,22) = 1.325, p = 0.286). Post hoc t-tests were used to explore the main effect of Condition, holding the level of each factor constant and investigating the effects of the other factor. These contrasts revealed a significant difference between Baseline condition and Spatially congruent condition (t(11) = −2.335, p = 0.040) and also between Baseline condition and Spatially incongruent condition (t(11) = −2.307, p = 0.042), but no significant difference between Spatially congruent condition and Spatially incongruent condition (t(11) = −0.058, p = 0.955). Note that correction for multiple comparisons is not generally recommended for the specific case of comparison between three conditions following significant omnibus ANOVA. Analysis of response bias (C values) showed no significant main effect of Condition (F(2,22) = 1.816, p = 0.186), or Side of tactile stimulation (F(1,11) = 3.794, p = 0.077), and no significant interaction between factors (F(2,22) = 2.385, p = 0.115).

## Discussion

The vestibular system has widespread interactions with other sensory modalities, including somatosensory signals. Multisensory neurons responding to vestibular and tactile stimulation were found in primate posterior parietal cortex (area VIP) [24], where the majority of the recorded cells encoded stimuli moving in the same direction. Another region in posterior parietal cortex (area 2v) immediately adjacent to primary somatosensory areas of hand and mouth also responds to vestibular stimulation coming from the semicircular canals and the otolith organs [27], [28]. Bimodal neurons coding for vestibular and tactile stimulation were also described in the so-called parieto-insular vestibular cortex (PIVC) [29] and such neurons responded to vestibular stimulation as well as touch applied on the arms, shoulders, neck, and legs. These findings were recently extended to humans: both caloric and galvanic artificial vestibular stimulation increased somatosensory sensitivity and modulated somatosensory potentials evoked by median nerve stimulation [8], [9], [10].

Here we observed that natural vestibular inputs elicited by passive whole-body rotation also enhanced tactile sensitivity. Importantly, this increase was independent from the spatial congruency between the direction of the rotation and the hand stimulated, since we found no evidence for a difference in the tactile sensitivity depending on whether the left or right finger received tactile stimulation during left or right passive whole-body rotations. Further, our data revealed that response bias is not affected by passive whole-body rotation. These results follow the predictions of a spatially non-selective vestibular-somatosensory interaction, and fail to follow the predictions of a spatially-selective vestibular-somatosensory interaction mediated by shifts in spatial attention or by spatially-selective perceptual mechanisms.

Vestibular stimulation has been often associated with shifts of spatial attention. Clinical reports in patients with circumscribed right hemispheric brain damage interpreted effects of artificial vestibular stimulation on tactile perception in terms of shifts of supramodal spatial attention toward the side of the space ipsilateral to the vestibular organs stimulated [13], [14]. Similarly, a recent study in healthy participants showed that vestibular stimulation by whole-body rotatory accelerations produces ipsiversive shifts of attention [18]. It is important to note that the experimental setup used in that study differed from the present study in important respects. First, the duration of rotation was much longer (6 s, compared to 2 s in the present study). Second, the stimuli were presented later during the acceleration phase (1500 ms after the beginning of rotation), than the stimuli in the present study. Our stimuli were presented at the peak of the acceleration phase (700 ms after the beginning of rotation). Third, the no-rotation interval between trials was much longer than in our experiment (15 s, in comparison to 6 s in the present study). Fourth, the tactile stimuli were well above threshold, whereas we used near-threshold stimuli. Fifth, Figliozzi et al. (2005) [18] asked participants to perform temporal order judgements rather than detection. Finally, the participants made manual responses whereas our study used unspeeded vocal responses. We can only speculate how all these various factors may influence the direct and indirect interactions between vestibular and somatosensory systems. However, we believe that the last point might explain the discrepancy between the results. Critically, Figliozzi et al. (2005) [18] used manual response keys placed along the direction of rotation. This was absent in our study, in which simple verbal responses were recorded. We therefore speculate that indirect mechanisms based on selective attention may dominate vestibular-somatosensory interactions when salient stimuli are processed and central motor plans are activated [18]. In contrast, direct vestibular-somatosensory interactions may be more important for perceptual processing close to threshold. In summary, our results cannot easily be reconciled with a spatially-selective attentional interpretation. Accounts based on *indirect,* attentional mechanisms would predict facilitatory effects on tactile detection only during spatially congruent rotations. Thus, during the present yaw rotation attention would be oriented toward the side of space and body congruent with the direction of the yaw rotation. However, our data did not reveal any difference between rotation directions both in tactile sensitivity and response bias.

In contrast, our study provides evidence for a direct vestibular-somatosensory interaction, independent of any modulation of rotation-dependant spatial attention or spatial perceptual mechanisms. Our results showed that natural vestibular stimuli elicited by passive whole-body yaw rotations produced an increase in tactile sensitivity similar to the effects described previously with artificial vestibular stimulations [8], [9]. Although the vestibular activations elicited by natural versus artificial vestibular stimulation are very different. At the peripheral level, the vestibular system is composed by three orthogonal semicircular canals detecting rotational movements of the head in the three-dimensional space (i.e., pitch, yaw and roll) and with two otolith organs (utricle and saccule) detecting translational acceleration, including the gravitational vertical. Artificial vestibular stimulations produce strong activations of both semicircular canals and otolith organs, while passive whole-body rotation as used here selectively stimulates the semicircular canals. Our results using yaw rotations suggest that the stimulation of canal-dependant rotational vestibular signals is sufficient to influence somatosensory processing.

Both somatosensory cortical areas and the insular cortex were found to respond to vestibular and somatosensory inputs in human neuroimaging studies, indicating an anatomical basis for the multisensory interaction between the two sensory modalities [3], [4], [5], [6]. We suggest that vestibular inputs could act to increase the firing of neurons responding to somatosensory input, thus enhancing somatosensory detection. Convergence of vestibular and tactile inputs onto bimodal neurons in these areas is one possible mechanism for this enhancement [30].

Caution is required in interpreting the non significant interaction that we found between direction of rotation and hand stimulated. Absence of interaction suggests that leftward and rightward rotations have similar effects on tactile sensitivity. This lack of lateralization is in contrast with previous findings using artificial vestibular stimulation, which found stronger somatosensory effects following vestibular stimulation designed to activate the vestibular network in the right hemisphere (i.e., left cold CVS [8]; left anodal and right cathodal GVS [9]). Neuroimaging studies using GVS identified the same asymmetry in the cortical vestibular system, suggesting that the cortical vestibular network is primarily located in the non-dominant right hemisphere in right-handed subjects [31]. However, the present data suggest that such hemispheric lateralisation induced by CVS and GVS might be related to the unusual unilateral nature of the artificial stimulation. During the natural rotatory stimulations used here, both left and right vestibular peripheral organs are activated, so that the input should be balanced across hemispheres. Thus, differences between the types of vestibular stimulation used and the consequent activations of vestibular afferents might explain the contrasting findings from artificial and natural vestibular stimulation. Natural vestibular stimulation produces balanced vestibular inputs to the two hemispheres, and shows spatially non-selective interactions with somatosensation. In contrast, existing methods of artificial vestibular stimulation involve a lateralised peripheral stimulus, both to the vestibular organs, and to other sensory receptors. For example, in many CVS studies, cold water is placed in the left ear. This not only activates the vestibular organ, but also provides a lateralised thermal and tactile stimulus. Spatially-selective effects of vestibular stimulation on other modalities might therefore, in principle, be due either to vestibular involvement in spatial attention, or to attentional effects of lateralised stimulation.

Could the enhancement in somatosensory sensitivity alternatively be an indirect effect of passive whole-body yaw rotation? For example, passive whole-body rotation might have increased general arousal. Our data cannot conclusively exclude this hypothesis. However, we believe an explanation based on arousal is unlikely for two reasons. First, some other sensory modalities such as vision [32] and nociception [33] are inhibited by artificial vestibular stimulation, in contrast to the facilitation of touch that we have reported. This speaks against a general arousal effect. Second, the natural vestibular stimulation in this experiment is similar to those encountered in everyday experience. Such natural head rotations do not seem to produce dramatic changes in arousal. However, further systematic investigation is required to investigate a possible role of arousal in vestibular-somatosensory interaction.

## Conclusion

Previous studies have focussed on the clinical [13], [14], anatomical [4], [6] and perceptual [8], [9] aspects of vestibular-somatosensory interactions as tested by unnatural vestibular stimulation. Here we show that naturally-evoked vestibular signals enhance near-threshold somatosensory processing. Our results are compatible with a direct and spatially non-selective modulation of somatosensory processing by concurrent vestibular input. Our results cannot readily be explained by changes in spatially-selective attention related to rotation.

## References

[1] Fasold O, von Brevern M, Kuhberg M, Ploner CJ, Villringer A, et al (2002) Human vestibular cortex as identified with caloric stimulation in functional magnetic resonance imaging. Neuroimage 17(3): 1384–1393. 12414278 10.1006/nimg.2002.1241

[2] Emri M, Kisely M, Lengyel Z, Balkay L, Márián T, et al (2003) Cortical projection of peripheral vestibular signaling. J Neurophysiol 89(5): 2639–2646. 12740408 10.1152/jn.00599.2002

[3] Zu Eulenburg P, Caspers S, Roski C, Eickhoff SB (2012) Meta-analytical definition and functional connectivity of the human vestibular cortex. Neuroimage 60(1): 162–169. 22209784 10.1016/j.neuroimage.2011.12.032

[4] Zu Eulenburg P, Baumgärtner U, Treede RD, Dieterich M (2013) Interoceptive and multimodal functions of the operculo-insular cortex: tactile, nociceptive and vestibular representations. Neuroimage 83: 75–86. 23800791 10.1016/j.neuroimage.2013.06.057

[5] Lopez C, Blanke O, Mast FW (2012) The human vestibular cortex revealed by coordinate-based activation likelihood estimation meta-analysis. Neuroscience 212: 159–179. 22516007 10.1016/j.neuroscience.2012.03.028

[6] Bottini G, Paulesu E, Sterzi R, Warburton E, Wise RJ, et al (1995) Modulation of conscious experience by peripheral sensory stimuli. Nature 376(6543): 778–781. 7651537 10.1038/376778a0

[7] Blanke O, Perrig S, Thut G, Landis T, Seeck M (2000) Simple and complex vestibular responses induced by electrical cortical stimulation of the parietal cortex in humans. J Neurol Neurosurg Psychiatry 69(4): 553–556. 10990525 10.1136/jnnp.69.4.553 PMC1737138

[8] Ferrè ER, Bottini G, Haggard P (2011) Vestibular modulation of somatosensory perception. Eur J Neurosci 34(8): 1337–1344. 21978189 10.1111/j.1460-9568.2011.07859.x

[9] Ferrè ER, Day BL, Bottini G, Haggard P (2013) How the vestibular system interacts with somatosensory perception: A sham-controlled study with galvanic vestibular stimulation. Neurosci Lett 550: 35–40. 23827220 10.1016/j.neulet.2013.06.046 PMC3988931

[10] Ferrè ER, Bottini G, Haggard P (2012) Vestibular inputs modulate somatosensory cortical processing. Brain Struct Funct 217(4): 859–864. 22466455 10.1007/s00429-012-0404-7

[11] Jung P, Baumgärtner U, Stoeter P, Treede RD (2009) Structural and functional asymmetry in the human parietal opercular cortex. J Neurophysiol 101(6): 3246–3257. 19357343 10.1152/jn.91264.2008 PMC3817274

[12] Eickhoff SB, Jbabdi S, Caspers S, Laird AR, Fox PT, et al (2010) Anatomical and functional connectivity of cytoarchitectonic areas within the human parietal operculum. J Neurosci 30(18): 6409–6421. 20445067 10.1523/JNEUROSCI.5664-09.2010 PMC4791040

[13] Vallar G, Sterzi R, Bottini G, Cappa S, Rusconi ML (1990) Temporary remission of left hemianesthesia after vestibular stimulation. A sensory neglect phenomenon. Cortex 26(1): 123–131. 2354638 10.1016/s0010-9452(13)80078-0

[14] Vallar G, Bottini G, Rusconi ML, Sterzi R (1993) Exploring somatosensory hemineglect by vestibular stimulation. Brain 116(1): 71–86. 8453466 10.1093/brain/116.1.71

[15] Kerkhoff G, Hildebrandt H, Reinhart S, Kardinal M, Dimova V, et al (2011) A long-lasting improvement of tactile extinction after galvanic vestibular stimulation: two Sham-stimulation controlled case studies. Neuropsychologia 49(2): 186–195. 21094654 10.1016/j.neuropsychologia.2010.11.014

[16] Schmidt L, Utz KS, Depper L, Adams M, Schaadt AK, et al (2013) Now you feel both: galvanic vestibular stimulation induces lasting improvements in the rehabilitation of chronic tactile extinction. Front Hum Neurosci 7: 90. 23519604 10.3389/fnhum.2013.00090 PMC3602932

[17] Lopez C, Blanke O (2011) The thalamocortical vestibular system in animals and humans. Brain Res Rev 67(1): 119–146. 21223979 10.1016/j.brainresrev.2010.12.002

[18] Figliozzi F, Guariglia P, Silvetti M, Siegler I, Doricchi F (2005) Effects of vestibular rotatory accelerations on covert attentional orienting in vision and touch. J Cogn Neurosci 17(10): 1638–1651. 16269102 10.1162/089892905774597272

[19] Angelaki DE, Cullen KE (2008) Vestibular system: the many facets of a multimodal sense. Annu Rev Neurosci 31: 125–150. 18338968 10.1146/annurev.neuro.31.060407.125555

[20] Schlack A, Sterbing-D'Angelo SJ, Hartung K, Hoffmann KP, Bremmer F (2005) Multisensory space representations in the macaque ventral intraparietal area. J Neurosci 25(18): 4616–4625. 15872109 10.1523/JNEUROSCI.0455-05.2005 PMC6725030

[21] Duhamel JR, Colby CL, Goldberg ME (1998) Ventral intraparietal area of the macaque: congruent visual and somatic response properties. J Neurophysiol 79(1): 126–136. 9425183 10.1152/jn.1998.79.1.126

[22] Henn V, Young LR, Finley C (1974) Vestibular nucleus units in alert monkeys are also influenced by moving visual fields. Brain Res 71(1): 144–149. 4206917 10.1016/0006-8993(74)90198-x

[23] Allum JHJ, Graf W, Dichgans J, Schmidt CL (1976) Visual-vestibular interactions in the vestibular nuclei of the goldfish. Exp Brain Res 26(5): 463–485. 1087607 10.1007/BF00238821

[24] Bremmer F, Klam F, Duhamel JR, Ben Hamed S, Graf W (2002) Visual–vestibular interactive responses in the macaque ventral intraparietal area (VIP). Eur J Neurosci 16(8): 1569–1586. 12405971 10.1046/j.1460-9568.2002.02206.x

[25] van Elk M, Blanke O (2012) Balancing bistable perception during self-motion. Exp Brain Res 222(3): 219–228. 22923207 10.1007/s00221-012-3209-2

[26] Macmillan NA, Creelman CD (1991) Signal Detection Theory: A User's Guide, New York, NY: Cambridge University Press.

[27] Büttner U, Buettner UW (1978) Parietal cortex (2v) neuronal activity in the alert monkey during natural vestibular and optokinetic stimulation. Brain Res 153(2): 392–397. 99209 10.1016/0006-8993(78)90421-3

[28] Guldin WO, Grüsser OJ (1998) Is there a vestibular cortex? Trends Neurosci 21(6): 254–259. 9641538 10.1016/s0166-2236(97)01211-3

[29] Grüsser OJ, Pause M, Schreiter U (1990) Localization and responses of neurones in the parieto-insular vestibular cortex of awake monkeys (Macaca fascicularis). J Physiol 430(1): 537–557. 2086773 10.1113/jphysiol.1990.sp018306 PMC1181752

[30] Avillac M, Hamed SB, Duhamel JR (2007) Multisensory integration in the ventral intraparietal area of the macaque monkey. J Neurosci 27(8): 1922–1932. 17314288 10.1523/JNEUROSCI.2646-06.2007 PMC6673547

[31] Dieterich M, Bense S, Lutz S, Drzezga A, Stephan T, et al (2003) Dominance for vestibular cortical function in the non-dominant hemisphere. Cereb Cortex 13(9): 994–1007. 12902399 10.1093/cercor/13.9.994

[32] Bense S, Stephan T, Yousry TA, Brandt T, Dieterich M (2001) Multisensory cortical signal increases and decreases during vestibular galvanic stimulation (fMRI). J Neurophysiol 85: 886–899. 11160520 10.1152/jn.2001.85.2.886

[33] Ferrè ER, Bottini G, Iannetti GD, Haggard P (2013) The balance of feelings: vestibular modulation of bodily sensations. Cortex 49(3): 748–758. 22385524 10.1016/j.cortex.2012.01.012

